# New Measurement Service for Determining Pressure Sensitivity of Type LS2aP Microphones by the Reciprocity Method

**DOI:** 10.6028/jres.116.019

**Published:** 2011-10-01

**Authors:** Randall P. Wagner, Victor Nedzelnitsky, Steven E. Fick

**Affiliations:** Mechanical Metrology Division, National Institute of Standards and Technology, Gaithersburg, MD 20899-8220

**Keywords:** acoustic measurements, acoustical calibration, calibration of microphones, electroacoustics, laboratory standard microphone, measuring microphone, microphone calibration, microphone pressure calibration, microphone reciprocity calibration, pressure reciprocity calibration of microphones, reciprocity calibration, standard microphones

## Abstract

A new National Institute of Standards and Technology (NIST) measurement service has been developed for determining the pressure sensitivities of American National Standards Institute and International Electrotechnical Commission type LS2aP laboratory standard microphones over the frequency range 31.5 Hz to 20 000 Hz. At most frequencies common to the new service and the old service, the values of the expanded uncertainties of the new service are one-half the corresponding values of the old service, or better. The new service uses an improved version of the system employed by NIST in the Consultative Committee for Acoustics, Ultrasound, and Vibration (CCAUV) key comparison CCAUV.A-K3. Measurements are performed using a long and a short air-filled plane-wave coupler. For each frequency in the range 31.5 Hz to 2000 Hz, the reported sensitivity level is the average of data from both couplers. For each frequency above 2000 Hz, the reported sensitivity level is determined with data from the short coupler only. For proof test data in the frequency range 31.5 Hz to 2000 Hz, the average absolute differences between data from the long and the short couplers are much smaller than the expanded uncertainties.

## 1. Introduction

Since the introduction of electroacoustical transducer calibration by the reciprocity method at the National Bureau of Standards in 1940 [1], this method has become established as the basis of the acoustic pressure measurement traceability chain. Modern realizations of this method [2, 3] are used to calibrate laboratory standard microphones designed for use as transfer standards, with critical electroacoustical properties and dimensions specified in U.S. national [4] and international [5] standards. For a sine-wave signal at each frequency of calibration, the calibration determines the modulus of the pressure sensitivity. This sensitivity is the amplitude ratio of microphone open-circuit output voltage to the sound pressure uniformly distributed over the diaphragm surface. The microphone sensitivity is usually expressed as the sensitivity level in decibels with respect to a reference of 1 V/Pa, and the measured sound pressure is usually expressed as the sound pressure level in decibels (reference: 20 μPa).

This paper introduces a new National Institute of Standards and Technology (NIST) measurement service for determining the pressure sensitivities of type LS2aP laboratory standard microphones [4, 5] over the frequency range 31.5 Hz to 20 000 Hz, and describes the equipment and procedures of this new service. Table 1 shows the expanded (coverage factor *k* = 2) uncertainties, expressed in decibels, for the old and the new NIST measurement services for pressure calibration of these microphones. The old service uses manually operated equipment to perform a comparison calibration of the customer microphone against each of two NIST-owned type LS1P laboratory standard microphones [4, 5] that are periodically calibrated by the reciprocity method and used as references.

Customers use the NIST-calibrated microphones to measure the sound pressure produced by instruments such as very stable sound calibrators, which are used to provide traceability for thousands of other acoustical measuring instruments used in critical measurements for product design and conformance, worker and military personnel safety, and health care. These instruments include measuring microphone systems, sound level meters, personal sound exposure meters (dosimeters), sound power measurement systems, and audiometric equipment.

Uncertainties for sound calibrator testing laboratories are established by U.S. national [6] and international [7] standards. The maximum permitted expanded (*k* = 2) uncertainty of measurement of the sound pressure level produced by the most accurate (Class LS) sound calibrators in laboratories at reference environmental conditions is 0.10 dB for the frequencies in Table 1 from 200 Hz to 1000 Hz. At these frequencies, the uncertainty of the new service is 0.06 dB smaller than the maximum permitted uncertainty.

The system used for the new service is an improved version of the automated test bed system used by NIST in the Consultative Committee for Acoustics, Ultrasound, and Vibration (CCAUV) key comparison CCAUV.A-K3 [8], and is now ready for the new NIST measurement service. The NIST results in CCAUV.A-K3 agreed well with the key comparison reference values. Subsequent improvements to this test bed system are:

1. control of the ambient static (barometric) pressure used for calibrations so that calibrations can be performed at the reference pressure specified in documentary standards [2–7] and key comparisons,
2. automated measurement and recording of the static pressure and acoustic coupler temperature at each frequency of measurement,
3. the use of the voltmeter in its most accurate alternating voltage measurement mode,
4. the use of new programmable highpass and lowpass filters to provide bandpass filtering with greater dynamic range and lower distortion than the previously used filter set,
5. an improved technique for measuring the front cavity depths of individual Type LS2aP microphones [9], and
6. adjustment of the power line frequency as needed to reduce interference effects at calibration frequencies harmonically related to 60 Hz [10].

## 2. Measurement System

**Fig. 1:**
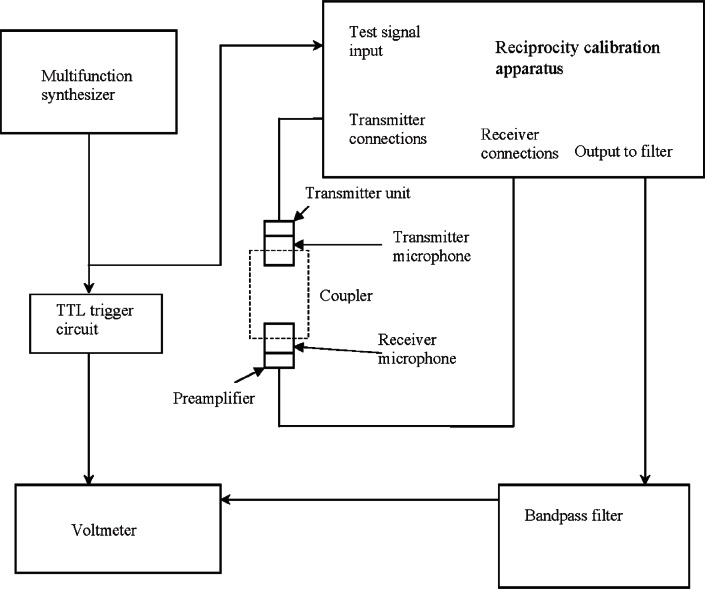
Block diagram of the microphone pressure reciprocity calibration system.

Reciprocity calibrations involve measurements on acoustically coupled pairs of microphones (see Fig. 1). Each pair includes a transmitter, which is electrically driven to produce sound, and a receiver, which produces an output voltage in response to the sound pressure at its diaphragm. These microphones are installed at opposed ports of an air-filled test cavity bounded by the microphone diaphragms and front-cavity walls, and the walls of a cylindrical plane-wave coupler. Two such air-filled couplers are used to perform reciprocity calibrations at NIST. Both couplers have an inner diameter equivalent to the diameter of an LS2aP microphone diaphragm. The first coupler, which has a length of 9.4 mm and will be referred to here as the long coupler, is a Bruel & Kjaer Type 1414.^1^ This coupler forms a nominal cavity volume of 700 mm^3^ with the microphones installed. The second coupler, which has a length of 4.7 mm and will be referred to here as the short coupler, is a Bruel & Kjaer Type 1430. It forms a nominal cavity volume of 400 mm^3^ with the microphones installed. Each coupler has a small tube passing through the coupler wall. This tube is partially plugged with a tapered pin and provides a vent for barometric pressure equalization during the measurements. Each microphone has a barometric pressure equalization vent from the back cavity of the microphone to its exterior outside the coupler. These coupler and microphone vents allow the barometric pressure on both sides of the diaphragm to be in equilibrium.

Laboratory standard microphones have a circular diaphragm that is recessed from a front outer annulus that is parallel to the diaphragm. The distance between the microphone diaphragm and the plane at the annulus is known as the front cavity depth. This depth is measured using a depth-measuring microscope according to gage block measurement subsampling method D [9]. The volume between the diaphragm and the plane at the annulus is known as the front cavity volume, which is about 34 mm^3^ for a Type LS2aP microphone. Because each microphone is installed at a coupler port, the front cavity volume is included in the total geometric cavity volume. An additional volume term that is used to calculate the microphone sensitivities is the equivalent diaphragm volume, which is related to the acoustic impedance of the microphone diaphragm [4, 5].

A block diagram of the microphone pressure reciprocity calibration system is shown in Fig. 1. At each frequency of measurement, a Hewlett Packard (HP) 8904A Multifunction Synthesizer supplies a sinusoidal test signal to a Bruel and Kjaer Type 5998 Reciprocity Calibration Apparatus (RCA). The RCA amplifies the test signal by 6 dB, and directs it to the transmitter through the transmitter unit, which contains a capacitor in series with the transmitter. Switches internal to the RCA direct either the receiver voltage signal path or the capacitor voltage signal path through an internal high-pass filter to the RCA output. The RCA output signal is passed through a bandpass filter to the voltmeter. The RCA amplifies the receiver and capacitor voltages by 40 dB, and also provides the 200 V polarizing voltage, which is required for the linear operation of the air-dielectric capacitive transmitter and receiver microphones.

The bandpass filter comprises cascaded Frequency Devices Model 90PF H8B and Model 90PF L8B programmable eight-pole highpass and lowpass Butterworth filters. For all measurements, the highpass filter corner frequency is set 8 % lower than the measurement frequency, and the lowpass filter corner frequency is set 8 % higher than the measurement frequency.

The output of the bandpass filter is connected to an HP 3458A Multimeter configured as a voltmeter in its mode that offers the highest accuracy for the measurement of periodic signals and requires a synchronous trigger. A trigger circuit that produces transistor-transistor-logic (TTL) pulses synchronized to the test signal is used to provide the voltmeter trigger. This arrangement is used to measure the amplified and filtered receiver and capacitor voltages, and the difference between the gains of the receiver and capacitor voltage signal paths. The synthesizer, RCA, bandpass filter, and voltmeter are controlled over an IEEE-488 bus by a personal computer running scripts with a Visual Basic script host.

The transmitter microphone is attached to a Bruel and Kjaer Transmitter Unit ZE 0796, and the receiver microphone is attached to a Bruel and Kjaer Preamplifier Type 2673. Both the transmitter unit and preamplifier incorporate Bruel and Kjaer Modification WH 3405, which conforms to the grounded shield configuration specified as the reference configuration in the relevant standards [2, 3, 4, 5]. The transmitter unit, receiver preamplifier, microphones, adapters, and coupler are installed in a Bruel and Kjaer Type UA 1412 Microphone Fixture with integral measurement chamber. This equipment isolates the coupled microphones from acoustical noise during the measurements.

An O-ring on the measurement chamber cover is used to seal the chamber shut, which allows for control of the measurement system barometric pressure. A small port on the bottom of the chamber is connected via tubing to an accumulator comprising two large insulated tanks, to minimize the effects of laboratory temperature fluctuations on the pressure. The second tank is connected to an air pump that is used to set the system to the desired pressure before electrical measurements are started with a given pair of microphones. The tanks and the measurement chamber are placed on a Technical Manufacturing Corporation Model 68–561 Vibration Isolation Table. Barometric pressure is measured with a Paroscientific 745-16B Barometer connected to a second small port on the bottom of the measurement chamber. A Hart Scientific 1529-R CHUBE4 Thermometer Readout is used with a Hart Scientific 5611A-CST Thermistor Probe to monitor the coupler temperature. Both the barometer and thermometer readings are acquired over the IEEE-488 bus. Because the barometer only has a RS232 port, a National Instruments GPIB-232CV-A, which is a GPIB-to-RS232 protocol converter, is used with the barometer on the bus. Relative humidity is measured with an RH Systems 473 Chilled Mirror Hygrometer System.

## 3. Data Acquisition

The reciprocity calibration procedure implemented at NIST is a primary method used to determine the sensitivities of three microphones in a triad comprising two NIST-owned microphones (microphones 1 and 2) and the customer microphone (microphone 3). Three pairwise combinations of transmitter and receiver are created from the triad.

Before starting the measurements, the microphone DC polarization voltage is adjusted as necessary to obtain a voltmeter reading between 199.999 V and 200.001 V, inclusive. Before data are acquired for a given pair, the pressure is adjusted to obtain a reading between 101.300 kPa and 101.350 kPa, inclusive, and the relative humidity is measured.

For each microphone pair, voltage data are acquired in order to determine the ratio of the receiver voltage to the capacitor voltage. Measurements are completed for all pairs in the long coupler from 31.5 Hz to 2000 Hz, and also in the short coupler from 31.5 Hz to 20 000 Hz. The barometric pressure and temperature are measured at each test frequency.

## 4. Data Reduction

The moduli of the pressure sensitivities of the three microphones |M*_p,_*_1_|, |M*_p,_*_2_|, and |M*_p,_*_3_|, when expressed in V/Pa, are given by the following equations based on standards [2, 3] and manufacturer’s technical documentation [11].

(1) $$\left|M_{p,1}\right|={\left|\frac{R_{12}\cdot R_{13}}{R_{23}}\cdot \frac{V_{0,12}\cdot V_{0,13}}{V_{0,23}}\cdot \frac{P_{S,23}}{P_{S,12}\cdot P_{S,13}}\cdot \frac{\kappa_{23}}{\kappa_{12}\cdot \kappa_{13}}\cdot \frac{Cor_{HW,12}\cdot Cor_{HW,13}}{Cor_{HW,23}}\cdot \frac{1}{C}\right|}^{1/2}$$

(2) $$\left|M_{p,2}\right|={\left|\frac{R_{12}\cdot R_{23}}{R_{13}}\cdot \frac{V_{0,12}\cdot V_{0,23}}{V_{0,13}}\cdot \frac{P_{S,13}}{P_{S,12}\cdot P_{S,23}}\cdot \frac{\kappa_{13}}{\kappa_{12}\cdot \kappa_{23}}\cdot \frac{Cor_{HW,12}\cdot Cor_{HW,23}}{Cor_{HW,13}}\cdot \frac{1}{C}\right|}^{1/2}$$

(3) $$\left|M_{p,3}\right|={\left|\frac{R_{13}\cdot R_{23}}{R_{12}}\cdot \frac{V_{0,13}\cdot V_{0,23}}{V_{0,12}}\cdot \frac{P_{S,12}}{P_{S,13}\cdot P_{S,23}}\cdot \frac{\kappa_{12}}{\kappa_{13}\cdot \kappa_{23}}\cdot \frac{Cor_{HW,13}\cdot Cor_{HW,23}}{Cor_{HW,12}}\cdot \frac{1}{C}\right|}^{1/2}$$

where the subscript *x* identifies the microphone number of the transmitter, and the subscript *y* identifies the microphone number of the receiver, *R_xy_* is the ratio of the receiver voltage to the capacitor voltage, *V_0,xy_* is the sum of the geometrical cavity volume and the low frequency equivalent diaphragm volumes of the microphones, *P_s,xy_* is the barometric pressure, *κ_xy_* is the ratio of specific heats of the air in the cavity, *C* is the capacitance of the capacitor in series with the transmitter, and *CorHW,xy* is a frequency-dependent parameter that accounts for the heat conduction and wave motion in the cavity as well as the frequency dependence of the equivalent diaphragm volumes.

The parameter *Cor_HW,xy_* includes a heat conduction correction that compensates for departures from purely adiabatic conditions due to heat exchange between the surfaces of the cavity and the air in the cavity. This correction, which increases with decreasing frequency, is a function of the cavity length to diameter ratio, volume to surface area ratio, and both the thermal diffusivity and the ratio of specific heats of the air. Wave motion effects are accounted for in *Cor_HW,xy_* by including a term that is dependent on the length of the cavity and the speed of sound in the cavity. This term is based on a homogeneous transmission line model used to evaluate the acoustic transfer impedance of the cavity. These effects are more pronounced at higher frequencies, where the wavelength of sound is smaller compared to the dimensions of the coupler.

To determine the front cavity volume of each microphone, an iterative fitting procedure is used. The value of the front cavity volume of each microphone is optimized in a two step procedure. The initial value for the first step is calculated from the measured front cavity depth and a nominal surface area. This value is varied in increments of 0.5 mm^3^ to find the minimum average absolute difference between the sensitivities determined for that microphone with the short coupler as compared to the long coupler at the eight frequencies measured in the range from 200 Hz to 2000 Hz. In this range the equivalent diaphragm volume is relatively constant with frequency, and the corrections for wave motion and heat conduction are relatively small for both couplers. Therefore, the match between the results for the two couplers is expected to be very good in this range. After the first step has been completed for all microphones, the results are used as initial values and the procedure is repeated to perform fits in increments of 0.05 mm^3^. The front cavity volume values obtained from these fits are used to calculate the final microphone sensitivities for both couplers.

For frequencies in the range from 31.5 Hz to 2000 Hz, the reported sensitivity is the average obtained from the results for both couplers. Above 2000 Hz, the reported sensitivity is the one obtained from results acquired with the short coupler only.

## 5. Uncertainties and Proof Tests

Uncertainties in the measured sensitivity levels of microphones calibrated by this new service were developed by applying guidelines for evaluating and expressing uncertainties [12]. Standard uncertainties and expanded (*k* = 2) uncertainties are shown in Table 2 for each frequency of measurement using the long coupler, and in Table 3 for each frequency of measurement using the short coupler. Relative uncertainties are expressed in percent. The standard uncertainties are rounded to the nearest 0.01 %. These uncertainties were combined to obtain the expanded (*k* = 2) uncertainties *U*, which were rounded up to the nearest 0.01 % and the nearest 0.01 dB. The Type B standard uncertainties correspond to the terms shown in Eq. (1–3) and are given in the tables. The standard uncertainties *u_P_* due to the terms denoted *P_s,xy_* are derived from the barometer manufacturer’s specifications. The standard uncertainties *u_C_* due to the *C*^−1^ term are derived from the transmitter unit manufacturer’s specifications. The standard uncertainties *u_R_* due to the terms denoted *R_xy_* are derived from the voltmeter manufacturer’s specifications and measurements of the electrical cross-talk, signal-to-noise ratios, and polarizing voltages in the calibration system. The standard uncertainties *u_V_* due to the terms denoted *V_0,xy_* are based on measurements of coupler dimensions and measuring instrument manufacturer’s specifications. The standard uncertainties *u_κ_* and *u_Cor_* due to the terms denoted *κ_xy_* and *Cor_HW,xy_* respectively, are based on general knowledge of the limitations of the models used to determine these terms.

The Type A standard uncertainties *u_A_* were determined from the results of proof tests conducted with seven microphones. For each microphone, six tests were conducted in both couplers. Each test was performed over 11 frequencies from 31.5 Hz to 2000 Hz for the long coupler, and 30 frequencies from 31.5 Hz to 20 000 Hz for the short coupler. At each of the 11 frequencies common to both couplers (31.5 Hz to 2000 Hz), there were 84 proof tests, 42 from each coupler.

For each frequency, coupler and microphone, the variance of the six measured sensitivities was calculated. For each frequency and coupler, the Type A standard uncertainty was calculated by pooling the variances for all seven microphones.

For each coupler, values of *U* are typically smallest for the frequencies near the middle of the frequency range, and are largest at the extremes. This variation with frequency is principally attributable to the frequency dependence of *u_Cor_*, the standard uncertainty in the parameter that accounts for heat conduction and wave motion effects in the cavity.

Table 4 shows the agreement between the results obtained for the long and short couplers at the frequencies where the data from both are averaged. At each frequency the absolute difference in the sensitivity levels determined using the long and short couplers was computed. The averages of the 42 absolute differences from all proof tests were determined, and are reported in Table 4 along with the expanded (*k* = 2) uncertainties for the new measurement service. These uncertainties are the results of rounding the average of the expanded uncertainties for both couplers upward to the nearest 0.01 dB. At all frequencies, the average absolute difference is much smaller than the expanded uncertainty. Therefore, the values of the microphone front cavity volumes are adequately determined by the iterative fitting procedure, and the significant influence factors that are different for the two couplers have been sufficiently taken into account.

## 6. Summary

This paper introduces a new NIST measurement service for determining the pressure sensitivities of ANSI and IEC type LS2aP laboratory standard microphones over the frequency range 31.5 Hz to 20 000 Hz, and describes the equipment and procedures used for this service. This service uses an improved version of the automated test bed system that was used for the NIST participation in the worldwide key comparison CCAUV.A-K3 [8].

Measurements are performed in a long and a short air-filled plane-wave coupler. An iterative fitting procedure varies the values of the front cavity volumes used to calculate the sensitivity of the microphones in order to achieve a match between the sensitivities determined using the long and the short couplers over the critical frequency range 200 Hz to 2000 Hz.

For each frequency in the range from 31.5 Hz to 2000 Hz, the reported sensitivity level for the new NIST measurement service is the average obtained from the results at that frequency for both couplers. For each frequency above 2000 Hz, the reported sensitivity level is the one determined from data acquired with the short coupler only. A series of proof test calibrations was performed to determine the Type A standard uncertainties. These uncertainties and the Type B standard uncertainties were used to obtain the expanded (*k* = 2) uncertainties.

As is shown in Table 1, the values of the expanded uncertainties of the new service are one-half the corresponding values of the old service, or better, at most frequencies common to the old and new services. This would particularly benefit customers who use the NIST service to meet the 0.10 dB maximum permitted uncertainty [6, 7] of measurement of the sound pressure level produced by Class LS sound calibrators from 200 Hz to 1000 Hz. At these frequencies, the uncertainty of the new service is now 0.06 dB smaller than the maximum permitted uncertainty.

A series of proof test calibrations was used to determine the average absolute differences between sensitivities measured with the long and the short couplers. At each frequency in the range 31.5 Hz to 2000 Hz, the average absolute difference is much smaller than the expanded uncertainty. Therefore, the values of the microphone front cavity volumes are adequately determined by the iterative fitting procedure, and the significant influence factors that are different for the two couplers have been sufficiently taken into account.

## Figures and Tables

**Table 1:** Comparison of old and new measurement service uncertainities

Frequency (Hz)	Old service expanded *k* = 2 uncertainty (dB)	New service expanded *k* = 2 uncertainty (dB)
31.5	Not offered	0.07
50	0.08	0.05
100	0.08	0.04
200	0.08	0.04
250	0.08	0.04
315	Not offered	0.04
500	0.08	0.04
700	0.08	0.04
1000	0.08	0.04
1500	0.08	0.04
2000	0.08	0.04
2500	0.08	0.04
3000	0.08	0.04
4000	0.08	0.04
5000	0.09	0.05
6000	0.09	0.05
7000	0.09	0.05
8000	0.26	0.06
9000	0.26	0.06
10 000	0.26	0.06
11 000	0.17	0.06
12 000	0.17	0.07
13 000	0.17	0.07
14 000	0.17	0.08
15 000	0.17	0.09
16 000	0.17	0.10
17 000	0.17	0.10
18 000	0.32	0.11
19 000	0.32	0.12
20 000	0.32	0.14

**Table 2:** Uncertainties for long coupler

Standard uncertainties (%)									
Frequency (Hz)	Type B						Type A	Expanded (*k* = 2) uncertainties	
	*u_P_*	*u_C_*	*u_R_*	*u_κ_*	*u_V_*	*u_Cor_*	*u_A_*	*U* (%)	*U* (dB)
31.5	0.01	0.04	0.06	0.06	0.13	0.32	0.09	0.74	0.07
50	0.01	0.04	0.05	0.06	0.13	0.17	0.09	0.50	0.05
100	0.01	0.04	0.05	0.06	0.13	0.11	0.08	0.42	0.04
200	0.01	0.04	0.05	0.06	0.13	0.10	0.08	0.41	0.04
250	0.01	0.04	0.04	0.06	0.13	0.10	0.08	0.41	0.04
315	0.01	0.04	0.04	0.06	0.13	0.10	0.07	0.40	0.04
500	0.01	0.04	0.04	0.06	0.13	0.10	0.07	0.40	0.04
700	0.01	0.04	0.04	0.06	0.13	0.10	0.08	0.41	0.04
1000	0.01	0.04	0.04	0.06	0.13	0.10	0.08	0.41	0.04
1500	0.01	0.04	0.05	0.06	0.13	0.10	0.08	0.41	0.04
2000	0.01	0.04	0.05	0.06	0.13	0.10	0.07	0.40	0.04

**Table 3:** Uncertainties for short coupler

Standard uncertainties (%)									
Frequency (Hz)	Type B						Type A	Expanded (*k* = 2) uncertainties	
	*u_P_*	*u_C_*	*u_R_*	*u_κ_*	*u_V_*	*u_Cor_*	*u_A_*	*U* (%)	*U* (dB)
31.5	0.01	0.04	0.06	0.06	0.13	0.32	0.10	0.75	0.07
50	0.01	0.04	0.03	0.06	0.13	0.17	0.08	0.49	0.05
100	0.01	0.04	0.03	0.06	0.13	0.11	0.08	0.41	0.04
200	0.01	0.04	0.03	0.06	0.13	0.10	0.11	0.43	0.04
250	0.01	0.04	0.03	0.06	0.13	0.10	0.07	0.39	0.04
315	0.01	0.04	0.03	0.06	0.13	0.10	0.10	0.42	0.04
500	0.01	0.04	0.03	0.06	0.13	0.10	0.07	0.39	0.04
700	0.01	0.04	0.03	0.06	0.13	0.10	0.12	0.44	0.04
1000	0.01	0.04	0.03	0.06	0.13	0.10	0.07	0.39	0.04
1500	0.01	0.04	0.04	0.06	0.13	0.10	0.08	0.41	0.04
2000	0.01	0.04	0.04	0.06	0.13	0.10	0.07	0.40	0.04
2500	0.01	0.04	0.04	0.06	0.13	0.11	0.08	0.42	0.04
3000	0.01	0.04	0.04	0.06	0.13	0.11	0.09	0.42	0.04
4000	0.01	0.04	0.04	0.06	0.13	0.11	0.10	0.43	0.04
5000	0.01	0.04	0.04	0.06	0.23	0.11	0.07	0.56	0.05
6000	0.01	0.04	0.04	0.06	0.23	0.11	0.09	0.57	0.05
7000	0.01	0.04	0.04	0.06	0.23	0.12	0.08	0.57	0.05
8000	0.01	0.04	0.04	0.06	0.23	0.13	0.10	0.59	0.06
9000	0.01	0.04	0.04	0.06	0.23	0.17	0.11	0.64	0.06
10000	0.01	0.04	0.04	0.06	0.23	0.21	0.07	0.66	0.06
11000	0.01	0.04	0.04	0.06	0.23	0.23	0.08	0.70	0.06
12000	0.01	0.04	0.04	0.06	0.23	0.25	0.09	0.73	0.07
13000	0.01	0.04	0.04	0.06	0.23	0.28	0.08	0.77	0.07
14000	0.01	0.04	0.04	0.06	0.23	0.32	0.08	0.83	0.08
15000	0.01	0.04	0.04	0.06	0.23	0.36	0.20	0.96	0.09
16000	0.01	0.04	0.04	0.06	0.23	0.39	0.32	1.13	0.10
17000	0.01	0.04	0.04	0.06	0.23	0.47	0.14	1.10	0.10
18000	0.01	0.04	0.04	0.06	0.23	0.55	0.15	1.25	0.11
19000	0.01	0.04	0.04	0.06	0.23	0.63	0.10	1.37	0.12
20000	0.01	0.04	0.04	0.06	0.23	0.71	0.12	1.53	0.14

**Table 4:** Average of 42 absolute differences in measured sensitivity levels determined using the long and short couplers, and expanded uncertainty for new NIST measurement service

Frequency (Hz)	Average absolute difference in sensitivity levels (dB)	Expanded (*k* = 2) uncertainty for new service (dB)
31.5	0.0079	0.07
50	0.0054	0.05
100	0.0033	0.04
200	0.0028	0.04
250	0.0013	0.04
315	0.0018	0.04
500	0.0006	0.04
700	0.0013	0.04
1000	0.0007	0.04
1500	0.0008	0.04
2000	0.0006	0.04

## Footnotes

1 Certain commercial equipment, instruments, or materials are identified in this paper to foster understanding. Such identification does not imply recommendation or endorsement by the National Institute of Standards and Technology, nor does it imply that the materials or equipment identified are necessarily the best available for the purpose.
